# The Crimean War

**Published:** 1910-01-01

**Authors:** Robert J. Banning


### THE CRIMEAN WAR.

### SOME MEDICAL AND SURGICAL REMINISCENCES.

By ROBERT J. BANNING, M.D., M.R.C.S., etc.

In the following article I have not attempted to describe in any detail the medical and surgical aspects of the Crimean War, but merely to relate some personal reminiscences. Hospital practice under war conditions has undergone such a complete change that all our arrangements at that time appear to us now to have been most crude and unsatisfactory. Modern science had scarcely begun to influence in any great measure medical treatment. Anæsthetics were quite in their infancy, and antiseptic treatment practically unknown.

Shortly after I had obtained the license of the Apothecaries' Society I interrupted my studies in order to take some part in the impending contest, and received an appointment in the transport service. I left London on November 18, 1854, and proceeded to Marseilles overland, where I joined the transport *Alps*, which embarked a battalion of Chasseurs à pied and some artillery. These troops were landed in good health at Kamiesch Bay, in the Crimea, the nearest point to the French camp, on December 19. I had during the whole of my service excellent opportunities for visiting the trenches, camps, and field hospitals and seeing the operations of the besieging batteries.

### CHRISTMAS IN THE CRIMEA.

On Christmas Day we received on board a number of English soldiers, mostly men wounded at the battle of Inkermann. Amongst them was Sir George Brown, K.C.B., the general commanding the Light Division. He was a Peninsular and Waterloo veteran, full of all the traditions of his old chief. A strict disciplinarian, he was by no means popular with his men. He insisted upon them marching, and incidentally fighting, in full uniform, with knapsacks, tight belts, and, above all, stiff leathern stocks. He had been shot through the arm, and the wounds were not healing kindly; the arm was also eczematous. However, by the time I handed over my charge at Malta he and the rest were nearly recovered. At this time and during the whole of my service no nurses were sent with the wounded and sick. Miss Nightingale and her too few companions had arrived, but although I saw her when she was prostrated with illness in the Crimea, I never in the transports had the benefit of any skilled nursing or medical assistance. I had to depend only on such help as imperfectly-trained orderlies could give. We certainly had the advantage of anæsthetics, but antiseptics were only occasionally available. Later on, owing to failure in supplies, chiefly due to storms and underestimating of quantities required, surgical appliances and medical comforts were only meagrely obtainable. Want of proper organisation, probably unavoidable, caused considerable quantities of stores to be overlooked in the confusion that reigned at Balaclava at the onset, and much was lost in the wreck of the s.s. *Prince* just as the need was greatest. Many weeks had to elapse before they could be replaced.

The *Alps* was sent to Marseilles again for French reinforcements. We returned to Kamiesch Bay on February 7, 1855. The French were as short of medical officers as our own forces, and I was appointed by the French Government surgeon to these troops. Until the 24th inst. I had good opportunities of witnessing the siege operations. Many days I passed in the trenches as a visitor, where casualties were constantly occurring. I had the pleasure of meeting Sir George Brown, now returned to his command, and also Admiral Sir Edmund Lyons and Admiral Bruat, respectively the English and French naval commanders. I paid a visit to Lord Raglan's headquarters, an oasis at this time in a sea of mud. I got on one occasion by favour into the advanced parallel, within 150 yards of the Redan, and able to see the principal objects of interest in Sebastopol. On February 20 there came a terrible blizzard, in which I was caught, at Balaclava, and I had to turn out with the others during the night, as the enemy came down on to the plain in considerable numbers, threatening the village. The attempt was abortive and no casualties ensued.

### FLORENCE NIGHTINGALE'S WORK.

Frost bites now became somewhat common, and I had several serious cases, some requiring amputations of fingers or toes. Soon after this we were ordered to embark invalided troops for home at Scutari Hospital; I was appointed to their medical charge by Dr. Cummings, P.M.O. I had an opportunity of going over the great hospital and seeing the work of Miss Nightingale and her little band of nurses. We received between two and three hundred on board, many of them very serious cases. Some I landed *en route* at Malta as being too ill to proceed further, but only one soldier died during the voyage. We had rough and trying weather most of the time, and reached Portsmouth on March 23. The Royal yacht *Fairy*, with the Queen, Prince Consort, and the Royal children on board, steamed quite close to the transport, and we mustered as many of the poor invalids on deck as possible who greeted Her Majesty with hearty though feeble cheers. Few as yet of the wounded had returned, and I think that the *Alps* with her melancholy freight was amongst the many noteworthy objects that met the Queen's eye that day.

### THE CHARGE OF THE LIGHT BRIGADE.

I again left for the Crimea on April 16, in medical charge of the transport *Cambria*, embarking Royal Artillery, men and horses, at Kingstown. These disembarked at Balaclava on May 4. This was the last time that I saw Lord Raglan, who came to see the reinforcements before landing. He died June 28 following. I had a good look over Balaclava Hospital, which I found had undergone considerable improvements. The enemy, too, having withdrawn their outposts, I was able to view the scene of the memorable charge of the Light Brigade. Many a mound, apparently of decaying animal matter, with horses' hoofs and shoes and debris of saddlery and clothing, scattered about. The siege operations had not progressed very brilliantly during the few weeks I had been away. There had been a great sortie from Sebastopol repulsed, and the French captured on the night of our arrival some rifle pits that had given trouble. There was by this time much improvement apparent in the camps, the tents in most divisions being superseded by wooden huts. The weather was becoming very hot, and some cases of cholera had occurred. I was glad, for hygienic reasons, when the *Cambria* was ordered out of the pestiferous Balaclava harbour to Kazatch Bay, almost close to Sebastopol. Here we were detained with steam up to take despatches from Lord Raglan to Malta. During the interval, by rowing cautiously in a small boat, we were able to get occasionally excellent peeps into the interior of the harbour of Sebastopol and of the seaboard fortifications. Again we took sick and wounded to Scutari Hospital, and, passing on, steamed to Malta as fast as possible, whence we returned to Balaclava with an immense quantity of ammunition of all kinds.

### Cholera.

On May 28 I was able to ride almost to the River Tchernaia, and also to see the Sardinian contingent, now beginning to arrive. I was able at this time to spend some days in the trenches, particularly in the Greenhill or Sailor's battery, and I also passed through the notorious Valley of Death. Cholera had now become epidemic. The few cases I had on board ship were successfully treated by the old-fashioned plan of calomel in somewhat large and frequently repeated doses, with chlorate or nitrate of potash in water as a drink, mustard plaisters, hot-water applications, and, essentially, constant friction of the cramped limbs. During the later stages port wine proved a good stimulant. With other medical officers, I found both brandy and opium valueless. In June I had medical charge of invalids for home, but returned immediately with a squadron of Carbineers and units of Scots Greys and Lancers with horses. A considerable number of these troops were landed at Scutari, as it was intended to limit the number of cavalry in the Crimea before the setting in of the second winter. In the Black Sea we suffered, the horses especially, great distress from the extreme heat and the quantities of locusts. flies, mosquitoes, and flying ants which infested the ship. Five days later we departed from Balaclava for Scutari with a large number of sick and wounded—English, French, and Sardinian officers —on board, in addition to 160 sick and undesirable Croatian mule drivers and camp followers. No interpreter was sent with these men, who were in a filthy condition, and wretchedly sea-sick. One died during the passage, and was promptly robbed by his fellows. They were landed at Constantinople, but most of the officers we took to Malta.

### In Sebastopol.

We arrived back at Balaclava on September 15, a week after the capture of the southern part of Sebastopol. As soon as I could I visited the captured town and made as thorough an inspection of the various forts, barracks, hospitals, and public buildings as I was able. The evidences of the terrific bombardment by the Allies were very striking. Everywhere a mass of ruins, scarcely a house that did not show signs of the destructive pounding they had undergone. The streets were strewn with *débris* of furniture, building materials, and even ammunition (including loose gunpowder) in large quantities. Many corpses were yet unburied, and in places the stench was intolerable. A day or two subsequently entry into the town was prohibited, owing to the danger from rifle fire across the narrow harbour. What struck me particularly was the enormous amount of unused cannon in the dockyard. Many of these are now war trophies in the principal cities of England and France. I visited the Redan, Malakoff Tower, Mamelon Verte and Karabalnœia Barracks, a dismal sight of ruin and decay. The hospitals, I saw, had all been evacuated and the patients removed to the opposite side. The bombardment of this, the northern portion, now commenced, but, owing to the change of front, involving new sap work and batteries, it proceeded but feebly and without any signal advantage. Soon afterwards I was sent to Sinope, in Asia Minor, and also to Kertch, in the Sea of Azoff, somewhat later.

### Final Days.

On October 11 the *Cambria*, in company with other transports and several frigates, sailed from Balaclava under sealed orders for Eupatoria, where a secret expedition consisting of 24 battalions of French and Turkish infantry, 38 squadrons of English, French, and Turkish cavalry, with 56 pieces of field artillery under the command of General d'Allonville, left on 27th inst. for Simferopol, the ancient capital of the Crimea. Our transport, towing H.M.S. *Diamond*, a sailing frigate, followed the course of the troops, marching by the sea shore. At the village of Sak they came upon the enemy strongly posted, and in the ensuing action drove them from their position. I was able to witness the whole of these operations, but all casualties were attended to on the field. We anchored off the Old Fort near the mouth of the River Alma. Two days later the force, having marched inland, returned, further advance having been found to be impracticable. Towing the frigate again to cover the army's rear from threatening Cossacks, we steamed slowly back to Eupatoria. Upon landing I found both men and animals in very sorry condition from the march through loose sand and over rocky ground. The Turks especially, never too well shod, were footsore and often bleeding, and all seemed utterly fagged out. We took some few wounded to Scutari hospital. We again returned to the Crimea, but, November being now almost ended, the weather was exceedingly bad and a constant succession of storms prevailed, during which many wrecks took place. I witnessed two or three at Eupatoria, where we were sent after vainly endeavouring to enter Balaclava harbour in the teeth of a gale. Returning thence after a few days we embarked at Balaclava a number of sick Turkish troops, with some English women and children and a number of our own officers and men for Scutari hospital. A fire broke out when about 60 miles at sea and some damage was done, but no panic ensued, as fortunately the women were all British. By this time I was suffering from an attack of, so-called, Crimean fever, and could only attend to my patients with difficulty and distress, so was not sorry to receive orders to take charge of convalescents for home. Calling at Malta on Christmas Day, we landed some and received others, and also a few more at Gibraltar, which place we left on New Year's Day, 1856. The invalids were disembarked at Plymouth. On January 6 Russia accepted basis of negotiations, and all hostilities ceased on the 16th inst. The treaty of peace was signed at Paris, March 30. I was much disappointed at not being accorded a war-medal for my services, although I received pay from the Admiralty. In subsequent campaigns they have been more freely bestowed.

